# Synthetic surfactin analogues have improved anti-PEDV properties

**DOI:** 10.1371/journal.pone.0215227

**Published:** 2019-04-11

**Authors:** Lvfeng Yuan, Shuai Zhang, Jie Peng, Yuchen Li, Qian Yang

**Affiliations:** 1 MOE Joint International Research Laboratory of Animal Health and Food Safety, College of veterinary medicine, Nanjing Agricultural University, Jiangsu, PR China; 2 College of veterinary medicine, Gansu Agricultural University, Gansu, PR China; Sun Yat-Sen University, CHINA

## Abstract

Surfactin has antiviral activity against various enveloped viruses by inhibiting viral membrane fusion. However, the potential utility of surfactin as an antiviral drug is limited by its cytotoxicity. In this study, 10 surfactin analogues were obtained by chemical synthesis and evaluated to determine their anti-PEDV activities, hemolytic activities, and critical micelle concentrations. The main goal of our study was to develop a safer drug; a surfactin analogue with high anti-PEDV activity and low hemolytic activity. Compared with surfactin, one of the analogues we developed, SLP5, has lower hemolytic activity, with the same antiviral activity. The selectivity index of SLP5 is 52, while the SI for surfactin is 4, in other words, the safe and effective concentration range of SLP5 is 12 times greater than that of surfactin. Like surfactin, SLP5 has a direct antiviral effect on PEDV. Structurally, SLP5 is a linear lipopeptide with three carboxyl groups. Surfactin derivatives similar to SLP5 could be obtained by lactone bond hydrolyzation of surfactin, as well as total synthesis.

## Introduction

Surfactin has antiviral activity against a variety of enveloped viruses, including herpes simplex virus (HSV-1, HSV-2), vesicular stomatitis virus (VSV), simian immunodeficiency virus (SIV) and Newcastle disease virus (NDV) [1, 2]. We recently demonstrated that surfactin exerts its antiviral effects by inhibiting viral membrane fusion [3]. Membrane fusion between the viral envelope and the cell membrane is essential for enveloped viruses to invade host cells. Surfactin can act directly on virus particles by insertion into the viral envelopes’ lipid bilayer and thereby reduce the membrane fusion rate. In addition, since the lipid components of viral envelopes are provided by the host cell, their composition, structure, and function are widely similar in the enveloped viruses. Surfactin has antiviral activity against numerous enveloped viruses, and it has promise as a broad-spectrum antiviral reagent, however, the effective dose range of surfactin is narrow, merely 4 x the antiviral concentration causes hemolysis and cytotoxicity. In this study we compared chemically synthesized surfactin analogues to determine future directions for surfactin modification.

Surfactin is a cyclic lipopeptide naturally produced by various strains of *Bacillus subtilis*, the structure consists of a seven amino acid peptide loop and a hydrophobic fatty acid chain. The production of designer surfactins, made by changing the number and composition of amino acids and fatty acids has proven to be an effective strategy for screening large numbers of lipopeptides for biological activity, but most current research focuses on their anticancer [4], antimicrobial [5] and insulin delivery [6] properties but not on their antiviral potential. Fatty acid chain length is critical to the antimicrobial effect of synthetic polymyxin analogues [7], while the specific antimicrobial spectrum of these analogues depends the amino acids in the lipopeptide [8]. Additionally, in synthetic daptomycin analogues, the introduction of aromatic groups in the fatty acid moiety also affects antibacterial activity [9]. In this study, a series of similar lipopeptides were designed and synthesized using surfactin as a template. These analogues differ from each other in the number of hydrophobic amino acids, number of hydrophilic groups, charge properties, amino acid chirality, position of hydrophilic amino acids, and aromatic groups in the fatty chain.

Porcine epidemic diarrhea virus (PEDV), a coronavirus, can infect pigs of all ages, but is especially virulent in newborn piglets, causing diarrhea, dehydration and even death [10]. Outbreaks of PEDV have been reported in many countries [11–14], and have caused immeasurable losses to the global swine industry. Recent research in our lab showed that surfactin acts directly on the PEDV envelope and inhibits the fusion process with the host cell membrane. In this study, PEDV was used as a target to screen chemically synthesized surfactin analogues for enhanced antiviral properties. Our results provide a theoretical basis for the development of new surfactin-derivative antiviral drugs.

## Material and methods

### Lipopeptide synthesis

All lipopeptides were synthesized by Synpeptide Co., Ltd (Shanghai, China). Mass spectrometry was used to determination molecular weight and to confirm product sequences. The purity of all synthetic lipopeptide samples was over 90% by HPLC. Ten synthetic lipopeptide samples were named SLP1 to SLP10. And their sequences as Table 1.

**Table 1:** Sequence of synthetic lipopeptide.

Name	Sequence
SLP1	Palmityl- L-Glu- L-Val- D-Leu- L-Ala- L-Asp- D-Leu- L-Val- NH_2_
SLP2	Palmityl- L-Glu- L-Val- D-Leu- L-Asp- D-Leu- L-Val- NH_2_
SLP3	Palmityl- L-Glu- L-Val- D-Leu- L-Asp- D-Leu- NH_2_
SLP4	Palmityl- L-Glu- L-Val- D-Leu- D-Leu- NH_2_
SLP5	Palmityl- L-Glu- L-Val- D-Leu- L-Asp- D-Leu
SLP6	Palmityl- L-Lys- L-Val- D-Leu- L-Lys- D-Leu- NH_2_
SLP7	Palmityl- L-Glu- L-Val- L-Leu- L-Asp- L-Leu- NH_2_
SLP8	Palmityl- L-Glu- L-Val- D-Leu- D-Leu- L-Asp- NH_2_
SLP9	Palmityl- L-Glu- L-Asp- L-Val- D-Leu- D-Leu- NH_2_
SLP10	Heptaalkyl-biphenyl-acid- L-Glu- L-Val- D-Leu- L-Asp- D-Leu- NH_2_

### Cells and virus

*Cercopithecus aethiops* kidney epithelial cells (Vero, ATCC, CCL-81) were cultured in high glucose DMEM (Gibco, US), supplemented with 10% fetal bovine serum (FBS, GIBCO), at 37 °C in a 5% CO_2_ humidified atmosphere. Cells were routinely seeded at a density of 2×10^5^ /mL in plastic tissue culture flasks (25 cm^2^ flasks, Corning, USA) and passaged every 3–4 days. PEDV CV777 was provided by the Jiangsu Academy of Agricultural Sciences (JAAS).

### Plaque reduction assay

Vero cells were seeded at 2 × 10^5^ cells/well in 24-well tissue culture plates and incubated 18–24 h at 37 °C until approximately 95% confluency was reached. 100 PFU of PEDV mixed with an equal volume of SLP in DMEM or DMEM alone were incubated 10 minutes at 37 °C, then added into the wells of the Vero cells and incubated 30 minutes at 4 °C. Cells were washed 3 times with DMEM then overlaid with DMEM/1% agar and incubated 72 hours at 37 °C. The cells were fixed with 4% formaldehyde, then stained with 0.1% crystal violet after the agar overlay was removed. The data are representative of 3 biological replicates and each plaque assay was performed in triplicate and values are expressed as means ± the standard deviation. Curve fitting and EC_50_ were calculated with GraphPad Prism 6 using the log[inhibitor] vs. response equation.

### Hemolytic assay

Hemolytic activity was measured according to the methods described in Jingdan [15], with some modifications. Briefly, serial dilutions of SLPs were added to 200 uL of a 1% suspension of porcine RBCs in PBS, followed by incubation for 1 h at 37 °C. Cells were centrifuged at 1000 g for 10 min, then 100 uL of each supernatant was transferred to wells of a 96-well plate. The optical density at 540 nm was measured using a microplate reader (Infinite 200 PRO, Tecan Group Ltd., Switzerland) and % hemolysis was calculated using the formula:

$$Hemolysis(\%)=\frac{{OD}_{S}-{OD}_{B}}{{OD}_{P}-{OD}_{B}}\times 100\%$$

where OD_S_, OD_B_, and OD_P_ represent the optical density of the SLP-treated samples, negative control and positive control, respectively. 1% Triton X-100 was used for the positive control. Each sample was run in triplicate.

### Critical micelle concentration

Surface tensions were measured using the ring method [16]. Samples were freshly prepared in a testing flask and allowed to stand for 30 minutes at 22 °C. Surface tension measurements were made using a surface tensiometer (HLD-LST-II, HENLIDA Co., Ltd, China). Linear regressions of the drop and the flat area were performed separately for the surface tension-concentration curve. The concentration at the intersection of the two lines is the critical micelle concentration (CMC).

### Time of addition assay

Time of addition assay was performed according to the procedures described [17], with modifications. Confluent Vero cells in 12-well plates were infected with 1000 PFU of PEDV and incubated for 1 hr at 4 °C to synchronize infection. The inoculum was removed and 1 mL of 37 °C DMEM was added to each well, cells were then placed at 37 °C in a humidified incubator. At the indicated time points, SLP5, surfactin, or dielaidoyl-phosphatidylethanolamine (DEPE, AvantiPolarLipids, Alabaster, AL) was added dropwise to cells or virus to a final concentration of 50 μg/ml, 20 μg/ml, or 5 μg/ml and incubated a further 12 hours at 37 °C. Viral nucleic acid and protein levels in the cells were measured by qRT-PCR and western-blot respectively. The data shown is representative of 3 independent experiments.

### RNA extraction and qRT-PCR

Total RNA was extracted from cells using TRIzol Reagent (Invitrogen) according to the manufacturer’s instructions. cDNA was generated by reverse transcription using HiScript TM QRT SuperMix for qPCR (Vazyme) according to the manufacturer’s instructions. PEDV nucleic acid levels were assessed by measuring the viral nucleoprotein (N) using qRT-PCR with the TaKaRa SYBR Green qPCR Kit (TaKaRa). Primer sequences were as follows: PEDV-N-F (sense), 5’-AAGGCGCAAAGACTGAACCC-3’; PEDV-N-R (antisense), 5’-TGTTGCCATTACCACGACTCC-3’; C-sabaeus-GAPDH-F, 5’-TCATCATCTCTGCCCCCTCT-3’; C-sabaeus-GAPDH-R, 5’-GTCATGAGTCCTTCCACGAT-3’. Gene level was calculated using the comparative Ct method and normalized to the endogenous levels of GAPDH.

### Western blot

Boiled cell lysates were subjected to SDS-PAGE then transferred to PVDF membrane (Roche, Basel, Switzerland) using a semi-dry transfer apparatus (GE, Little Chalfont, Buckinghamshire, UK). Membranes were blocked in 5% non-fat milk in TBS containing 0.01% Tween-20 (TBST) then incubated overnight at 4 °C with anti-PEDV N-protein monoclonal antibody (Median Diagnostics, South Korea) diluted in TBST with 1% BSA. Membranes were washed 30 min in TBST followed by incubation for 1 h at RT with 1:5000 HRP goat anti-mouse IgG antibody (ABGENT, San Diego, CA, USA) then thrice washed for 15 min in TBST. Membranes were incubated with ECL reagent (Thermo, Waltham, MA, USA) and imaged using a ChemiDoc™ system (Bio-Rad, Hercules, CA, USA).

## Results

### Lipopeptide design

**Fig 1:**
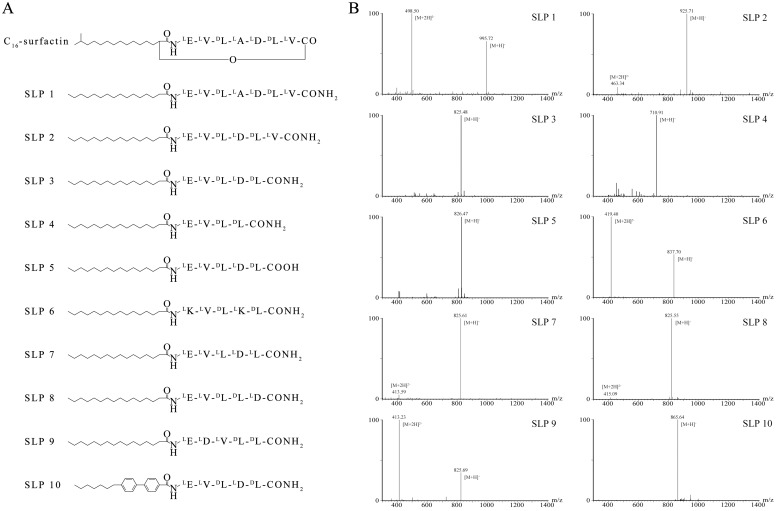
Synthesis of surfactin analogues. (A) Chemical structures of surfactin and novel synthetic lipopeptides. The peptides are indicated using IUPAC single-character symbols, and the superscripts indicate amino acid chirality. (B) Mass spectra of synthetic surfactant analogues.

Native surfactin is an amphiphilic cyclic lipopeptide, it consists of a heptadine interlinked with fatty acid. Using the native structure as a template, we designed 10 surfactin analogues that were then commercially synthesized (Fig 1A). SLP1 consists of a heptadiene identical to the peptide of native surfactin, and a fatty acid chain the same length as in native surfactin. The N’ of heptadine is linked to the fatty acid and the C'- is aminated. SLP2 and SLP 3 have one and two fewer hydrophobic amino acids respectively, than SLP1. Relative to SPL3, SLP4 has one fewer hydrophilic amino acids, while SLP5 has a free carboxyl at its C'-end that acts as an additional hydrophilic group. SLP6 replaces the two acidic amino acids of SLP 3 with lysine (a basic amino acid). SLP7 is a version of SLP3 that contains only L-amino acids. The two hydrophilic amino acids of SLP8 are distant from each other, but are in close proximity in SLP9. SLP 10 is identical to SLP3 except for a diphenyl group in the fatty acid moiety. The mass spectra of the SLPs are shown in Fig 1B and are consistent with the expected molecular weights. In all cases, SLP purity exceeded 90%.

### Anti-PEDV activity of the lipopeptides

**Fig 2:**
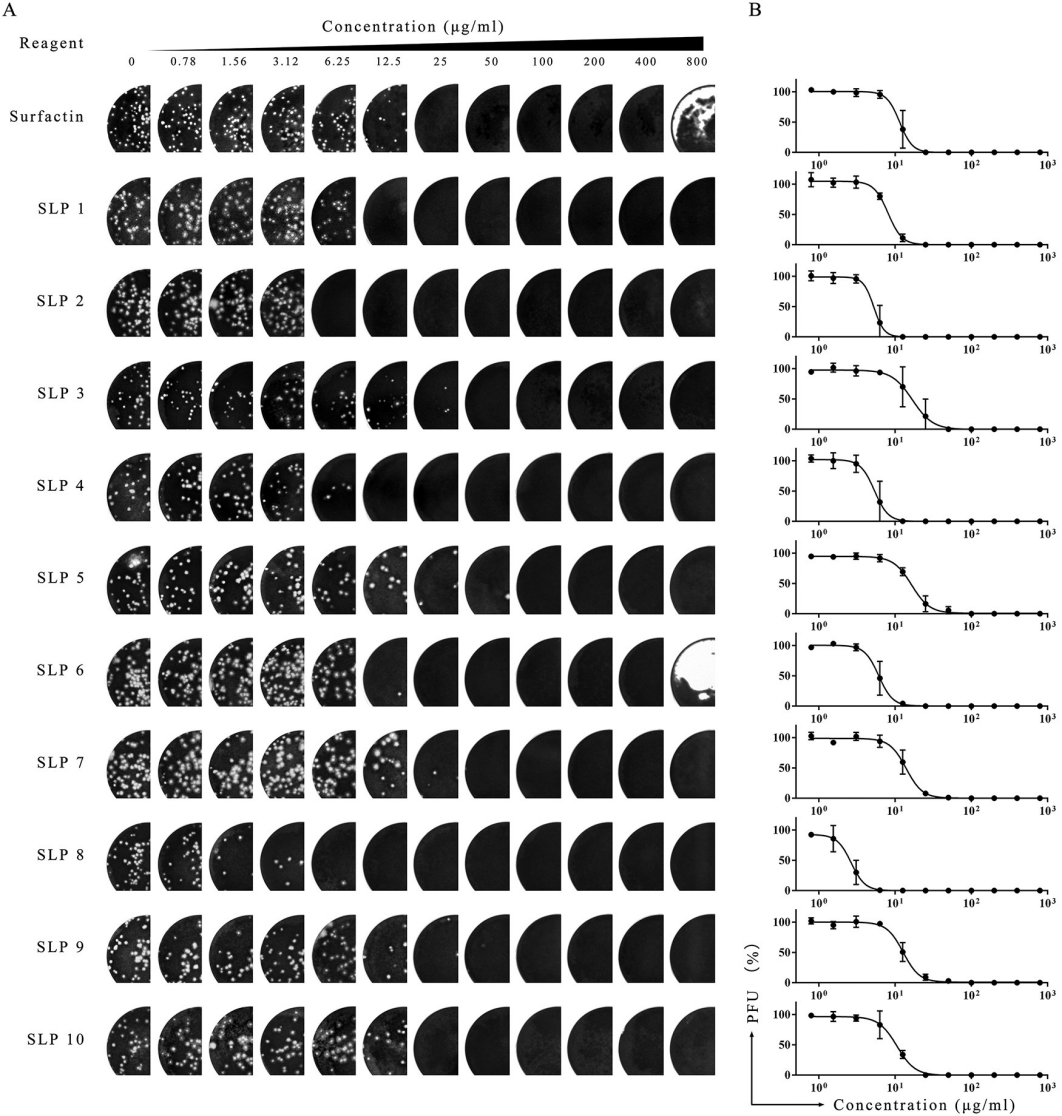
Anti-PEDV activities of lipopeptides. (A) PEDV was incubated with series concentrations of SLPs for 10 minutes at 37 °C. After washing cells of unadsorbed virus, the remaining infectious particles was detected by plaque assay. (B) Anti-PEDV activity (% remaining PFU) plotted as a function of lipopeptide concentration. The results of three independent experiments are shown as means ± standard deviation. 100% represents plaque counts in the control (no lipopeptide) sample.

The SLPs were tested for anti-PEDV activity using a plaque reduction assay. Serial dilutions of SLPs were incubated with an equal volume of PEDV then aliquoted onto precooled cells in 12-well plates. After the virus has adsorbed to the cells at 4 °C, SLPs were washed away. The adsorbed virus particles were then counted by plaque forming assays (Fig 2A). The results were fitted to a sigmoid-curve and plotted Fig 2B. All SLPs have potent anti-PEDV effects above 50 μg/ml, but differences in their anti-PEDV effect. SLP2, SLP4, SLP6, and SLP8 have a higher potency than surfactin.

### Hemolytic activity of lipopeptides

**Fig 3:**
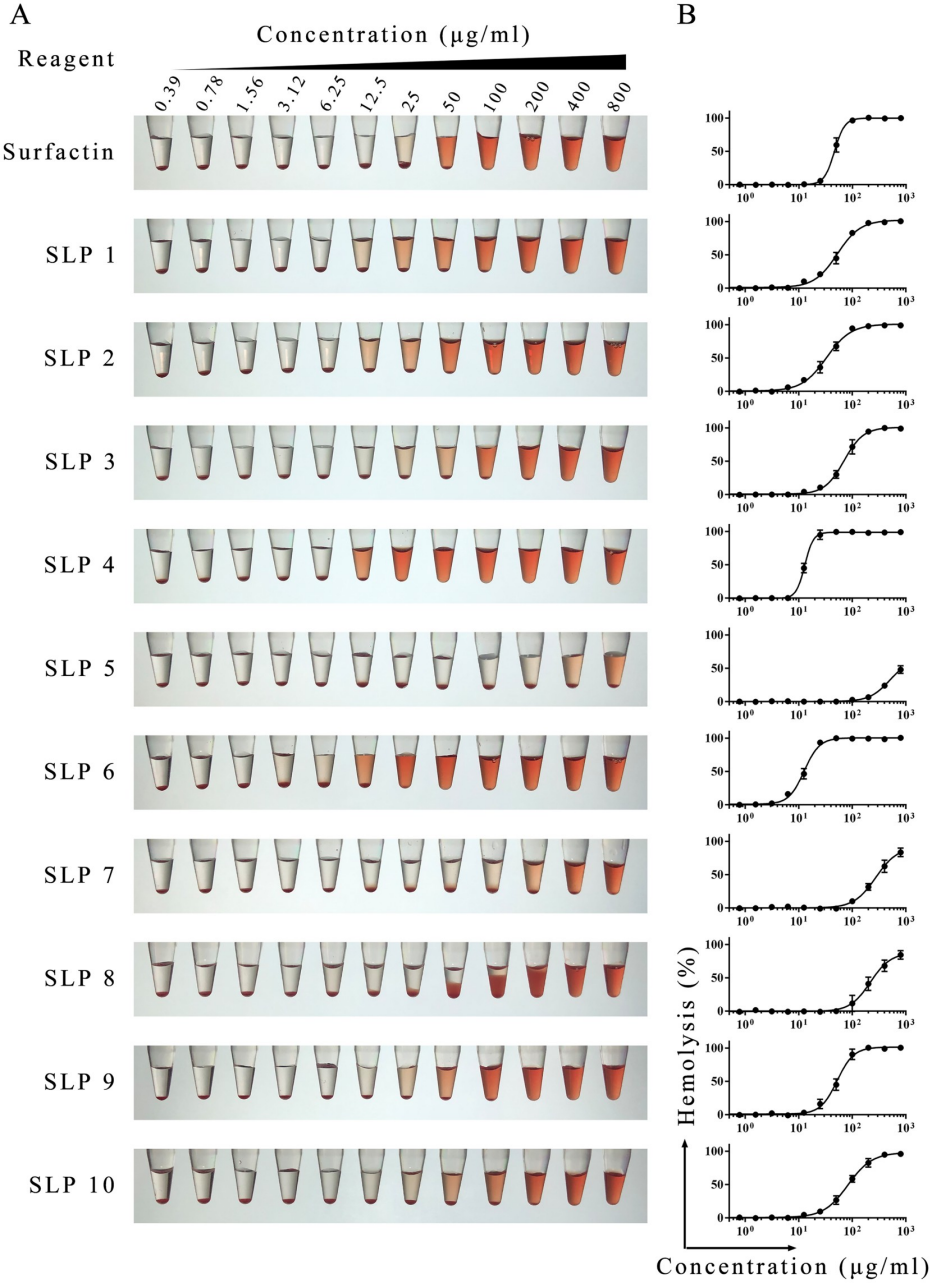
Hemolytic activity of lipopeptides. (A) The indicated concentration of SLPs were added to a 1% porcine RBC suspension in PBS, followed by incubation for 1 h at 37 °C and centrifugation. Pictures were taken after collecting a portion of the supernatant for quantification of hemolysis. (B) Hemolytic curve. Hemolysis (measured at OD_540_) is shown as a function of SLP concentration. 100% represents the OD obtained using 0.1% Triton X-100-treated red blood cells.

Hemolysis is commonly used to assess the biosafety of lipopeptides [18, 19]. As shown in Fig 3, SLPs have a range hemolytic activities. Those of SLP1, SLP2, SLP3, and surfactin are similar, suggesting that the number of hydrophobic amino acids has little effect on hemolytic activity. However, other modifications significantly affect activity; SLP4, SLP3 and SLP5 have one, two and three carboxyl groups respectively, and the hemolytic activity is reduced in turn. In addition, the chiral differences that distinguish SLP7 and SLP3 at two amino acid residues, and the differences in the arrangement of amino acids between SLP8 and SLP9 also affect hemolytic activity. These results indicate that amino acid composition by itself does not completely determine the characteristics of SLPs. It is noteworthy that SLP8 has an unexpected effect on porcine erythrocytes. Although SLP8 has a low hemolytic activity, it prevents the precipitation of red bloods under our assay conditions at concentrations above 50 μg/ml.

The selectivity index (SI) was determined as the ratio of the 50% cytotoxicity concentration (CC_50_) to the 50% effective concentration (EC_50_). As shown in Table 2, SLP8 and SLP5 have the highest and second-highest SI values respectively. Given the anti-sedimentation effect of SLP8 on porcine blood cells, SLP5 was selected as the most promising candidate. Although SLP4 and SLP6 have stronger antiviral activity than surfactin, their SI is lower due to their high hemolytic activity.

**Table 2:** Biological activities of surfactin and its analogues.

Name	EC_50_ (μg/ml)	CC_50_ (μg/ml)	SI^a^
Surfactin	11.4 ± 0.7	45.9 ± 0.9	4.0
SLP1	8.0 ± 0.3	52.6 ± 2.3	6.5
SLP2	5.3 ± 0.5	33.0 ± 1.6	6.3
SLP3	16.9 ± 1.6	69.7 ± 2.6	4.1
SLP4	5.4 ± 0.4	12.9 ± 0.2	2.4
SLP5	16.5 ± 0.6	847.2 ± 124.9	51.5
SLP6	6.1 ± 0.3	12.6 ± 0.4	2.1
SLP7	14.0 ± 0.6	274.1 ± 21.0	19.7
SLP8	2.6 ± 0.2	217.5 ± 18.9	82.1
SLP9	12.8 ± 0.5	52.2 ± 2.0	4.1
SLP10	10.6 ± 0.5	82.8 ± 3.4	7.8

^a^ Selectivity index, SI = CC_50_/EC_50_.

### Determination of critical micelle concentrations

**Fig 4:**
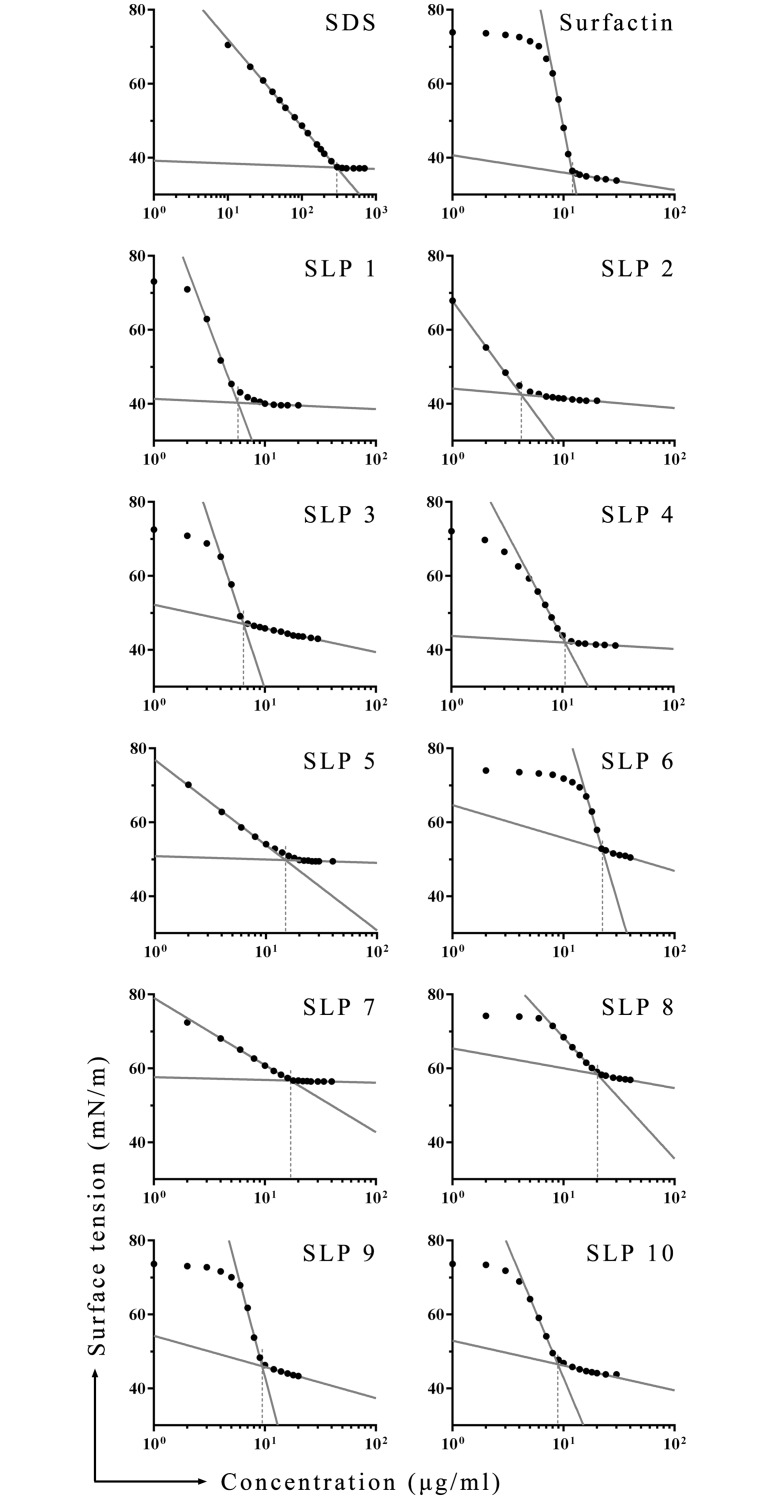
Critical micelle concentrations of the SLPs. CMCs were determined for SDS, surfactin, and SLPs by surface tension. The intersection of the two linear regression lines corresponds to the CMC value, (shown by the dotted lines).

The CMC was calculated by measuring the surface tension of each SLP over a range of concentrations. As shown in Fig 4, surface tension decreases as SLP concentration increases, and as it approaches its lowest value, the curve exhibits an inflection point when the SLP concentration reaches CMC. Linear regressions were performed independently using the data on the descending part of the curve below the CMC and the equilibrium part above the CMC. The intersection of the linear regression lines corresponds to the CMC (Table 3).

**Table 3:** Critical micelle concentration values.

Name	CMC (μg/ml)	Name	CMC (μg/ml)
SDS	296	SLP5	15.0
Surfactin	12.1	SLP6	22.4
SLP1	5.7	SLP7	17.1
SLP2	4.2	SLP8	20.2
SLP3	6.4	SLP9	9.4
SLP4	10.6	SLP10	8.9

**Fig 5:**
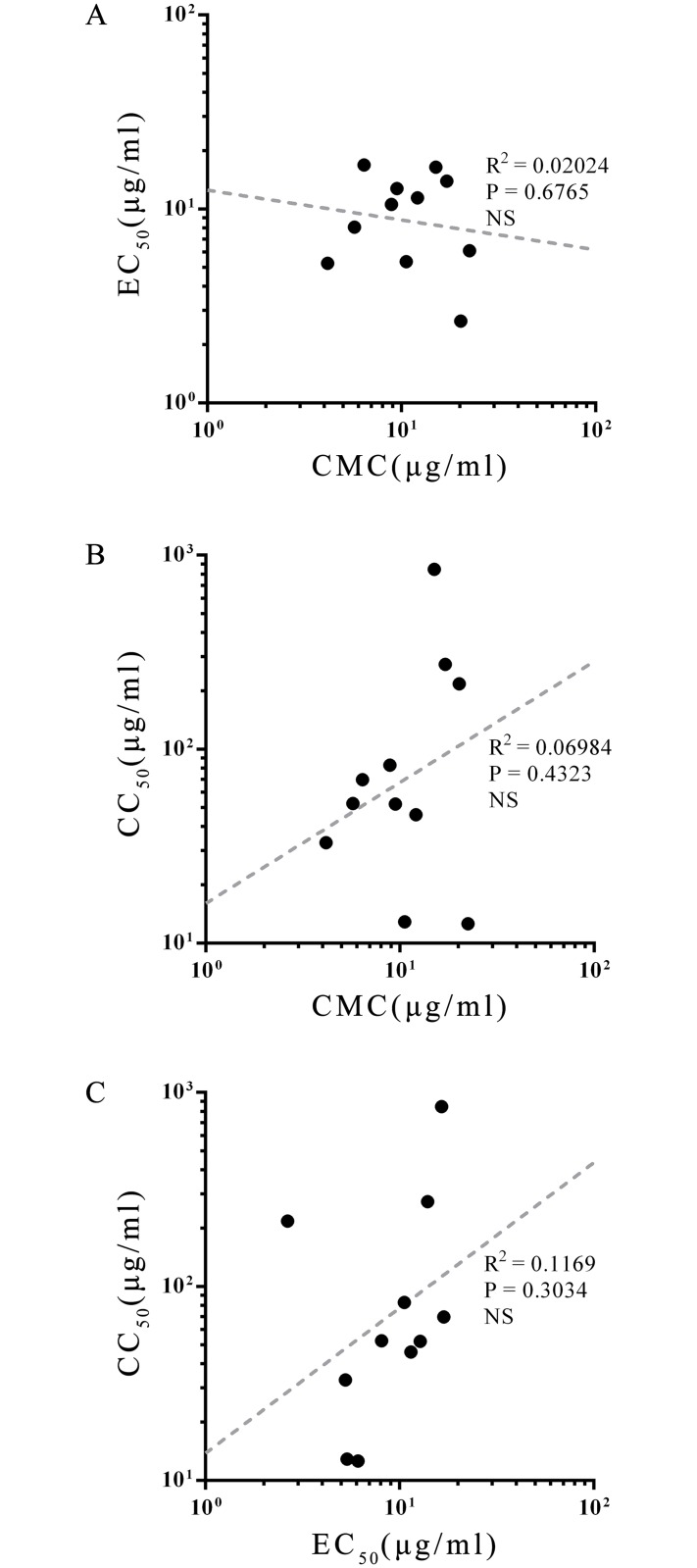
Correlation of SLPs eigenvalues. Scattergrams of (A) EC_50_-CMC, (B) CC_50_-CMC, and (C) CC_50_-EC_50_ for surfactin and SLPs. The dotted lines show the results of linear regression for each scattergram, along with R^2^ and p values. None of the correlations are significant.

In order to explore the relationship between CMC, anti-PEDV activity, and hemolytic activity, scatter plots for pairs of assays (Fig 5). Each plot contains 11 points representing the characteristic indices of SLP1 to 10 and surfactin. The distribution of points has no obvious regularity in any of the three plots, and the linear regression results are not significant. We conclude that the anti-PEDV and hemolytic activities of the SLPs examined in this study are not related to their surfactant activity (represented by the CMC). In addition, there is no significant correlation between anti-PEDV activity and hemolytic activity. Synthetic lipopeptides that combine greater antiviral activity with lower cytotoxicity are still to be found, and the specific structure-activity relationship needs to be further elucidated.

### SPL5 acts directly on PEDV

**Fig 6:**
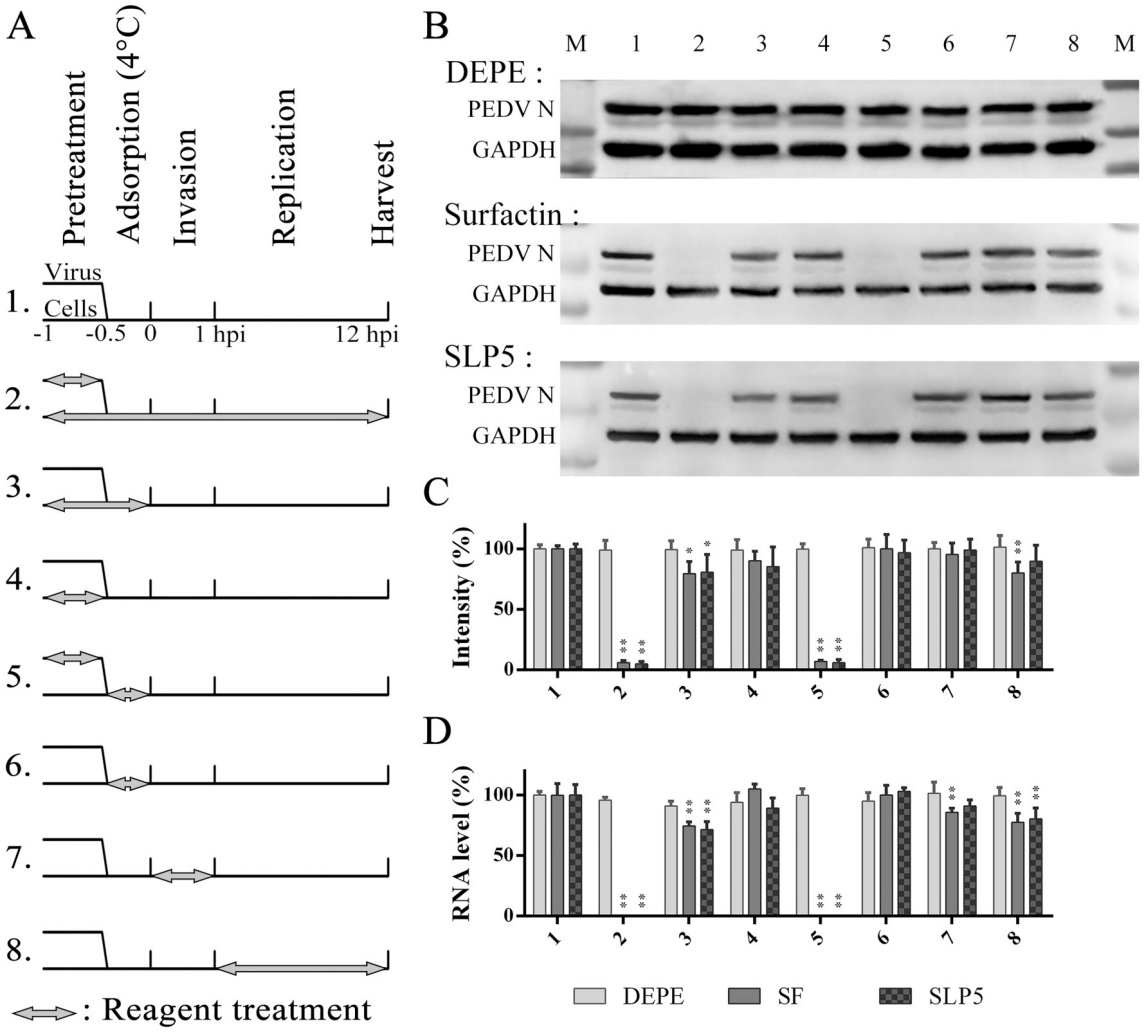
Time-of-addition experiments. (A) Vero cells were treated with SLP5, surfactin, or DEPE at different time points before, during, and after infection. The presence of the reagent is indicated by arrows. The treatment group number is on the left of the figure. Samples were harvested at 12 hpi. (B) The PEDV nucleoprotein was detected by Western-blot, and (C) quantitated by band intensity relative to the internal control, GAPDH. (D) The PEDV genome was detected by qRT-PCR. The experiment was repeated three times, normalized against Group 1, and plotted as mean ± SD. *, p < 0.05; **, p < 0.01 in two-tail t-test, for each reagent, compared with Group 1.

Since SLP5 was the most promising surfactin analogue, we chose it for further study. Time of addition assays were performed to determine whether the SLP5 exerts its anti-PEDV effect at the same stage during infection as surfactin. Since surfactin acts directly on virions, the experiment was designed to examine events early in the infection process. Fig 6A summarizes the experimental plan and the eight treatments tested. Briefly, SLP5, surfactin, or DEPE was added to virus alone, cells alone, or to cells and virus together prior to infection, during virus adsorption, during virus invasion (1 h post infection at 37 °C), or during replication (1–12 hpi). Samples were harvested 12 hours after infection, and analyzed to measure cellular levels of viral protein (Fig 6B and 6C) and viral RNA (Fig 6D). As expected for a normal component of the cell membrane, DEPE did not affect PEDV replication at any stage, while SLP5 and surfactin exhibited antiviral activity at specific stages. For example, when SLP5 or surfactin are present throughout the experiment (treatment group 2), little PEDV replication occurs, as indicated by nucleic acid and protein levels. The same is true in treatment group 5, indicating that both compounds act directly on the virus. Other antiviral mechanisms of SLP5 and surfactin cannot be ruled out since PEDV replication is inhibited to different degrees in some of the other treatment groups.

## Discussion

Wedge-shaped lipids in which the hydrophilic head has a larger cross-sectional area than the hydrophobic tail, are potential membrane fusion inhibitors [20, 21]. In a recent study of rigid amphipathic fusion inhibitors, compounds with deoxyribose or acetate as hydrophilic moieties had anti-HSV effects [22]. In addition, a recent study on antimicrobial activity of synthetic lipopeptides reported that lipopeptides with two to four positive charges and 16 carbon atoms in the lipid chain have potent antimicrobial activity [23]. Fatty acid chain length from 8 to 16 carbon atoms is positively correlated with antimicrobial activity, but is also positively correlated with hemolytic activity and membrane selectivity [24]. These factors must be considered in the design of new lipopeptides, and the structure-function relationships warrant further study. Here we investigated lipopeptides containing two and three negative charges, and two positive charges, all with a fatty acid chain of 16 carbon atoms. All of these lipopeptides had antiviral activity.

In a previously published study we demonstrated that surfactin has antiviral activity as membrane fusion inhibitor [3]. In order to improve the viral envelope selectivity of surfactin, we designed 10 analogues with altered peptide amino acids, but kept the length of fatty acid chain of 16 carbon atoms. Compared with SLP3, SLP5 has one additional carboxyl group at the end of the peptide, but the antiviral effect is similar. Although SLP4 has one less hydrophilic amino acid than SLP3, its antiviral activity increases about 3-fold. This result indicates that the hydrophilic portion of the wedge-shaped lipid needs to be sized appropriately for better antiviral function. In addition, SLP8 differs from SLP3 only in the order of two amino acids, but the antiviral potency of SLP8 is 6.5 times greater, indicating the complexity of the relationship between molecular structure and antiviral effects.

The cationic properties of synthetic lipopeptides are thought to be related to their antibacterial and hemolytic activities [25]. SLP6, the only cationic lipopeptide examined in this study, has the strongest hemolytic activity, consistent with this hypothesis. Cyclic lipopeptides are reported to more readily lyse bacterial membranes than linear or branched lipopeptides [26]. In agreement, we found that the hemolytic activity of SLP1 (a linear molecule, identical in amino acid sequence to surfactin) is slightly weaker than that of surfactin, which has a cyclic structure. It has also been reported that the total hydrophobicity of synthetic lipopeptides is inversely related to hemolytic activity, although this conclusion was from data on only 3 related samples [27]. Our results do not support this conclusion. The relative hydrophobicity of SLP1, 2, and 3 is SLP1>SLP2>SLP3, as indicated by their HPLC retention times (17.6, 16.7, and 14.5 min, respectively). However, their hemolytic activity is not significantly related to the total hydrophobicity.

In summary, we synthesized 10 surfactin analogues and characterized them to identify those with enhanced anti-viral properties. SLP5 equaled and SLP8 exceeded surfactin’s anti-PEDV activity and both compounds had greatly reduced hemolytic activity, making their selection indexes 13 and 21 times larger respectively than surfactin’s. However, SLP8 exhibited a peculiar inhibition of red blood cell sedimentation. SLP5 is therefore a more promising synthetic surfactin analogue. Compared with surfactin, the structure of SLP5 is distinguished by its linear lipopeptide and the additional carboxyl group at the C' of the peptide. SLP5 also has two fewer hydrophobic amino acids than surfactin, this reduces the cost of synthesis while having little effect on antiviral activity. Since surfactin has been shown to protect piglets from PEDV challenge [3], the *in vivo* antiviral properties of surfactin analogues needs to be tested. Furthermore, with respect to the future use of synthetic surfactins to control or ameliorate infection in pigs, the broader spectrum physiological activities of surfactin analogues needs further study and side effects beyond hemolytic toxicity need to be explored. Our study suggests that SLP5, and other surfactin analogues, should be the object of in-depth study in order to develop a safer broad-spectrum family of surfactin antiviral agents.
